# Ultrasonic RF time series for early assessment of the tumor response to chemotherapy

**DOI:** 10.18632/oncotarget.23625

**Published:** 2017-12-23

**Authors:** Qingguang Lin, Jianwei Wang, Qing Li, Chunyi Lin, Zhixing Guo, Wei Zheng, Cuiju Yan, Anhua Li, Jianhua Zhou

**Affiliations:** ^1^ Department of Ultrasound, Sun Yat-Sen University Cancer Center, State Key Laboratory of Oncology in South China, Collaborative Innovation Center for Cancer Medicine, Guangzhou, 510060, P.R. China; ^2^ School of Electronic and Information Engineering, South China University of Technology, Guangzhou, 510640, P.R. China

**Keywords:** chemotherapy, cancer, ultrasonic radio-frequency time series, microstructure

## Abstract

Ultrasound radio-frequency (RF) time series have been shown to carry tissue typing information. To evaluate the potential of RF time series for early prediction of tumor response to chemotherapy, 50MCF-7 breast cancer-bearing nude mice were randomized to receive cisplatin and paclitaxel (treatment group; n = 26) or sterile saline (control group; n = 24). Sequential ultrasound imaging was performed on days 0, 3, 6, and 8 of treatment to simultaneously collect B-mode images and RF data. Six RF time series features, *slope, intercept, S1, S2, S3*, and *S4*, were extracted during RF data analysis and contrasted with microstructural tumor changes on histopathology. Chemotherapy administration reduced tumor growth relative to control on days 6 and 8. Compared with day 0, *intercept, S1*, and *S2* were increased while *slope* was decreased on days 3, 6, and 8 in the treatment group. Compared with the control group, *intercept, S1, S2, S3*, and *S4* were increased, and *slope* was decreased, on days 3, 6, and 8 in the treatment group. Tumor cell density decreased significantly in the latter on day 3. We conclude that ultrasonic RF time series analysis provides a simple way to noninvasively assess the early tumor response to chemotherapy.

## INTRODUCTION

Cancer has become a huge burden for our society. According to the GLOBOCAN 2012 estimates, there were about 14.1 million newly diagnosed cases of cancer and 8.2 million cases of cancer-related deaths in the world [1]. Although more than half of the cancer patients receive the treatment of cytotoxic agents, marked tumor heterogeneity leads to variable responses even for tumors with the same staging and cytopathologic characteristics. Thus, it is crucial to accurately evaluate early tumor response to treatment in order to prevent unnecessary chemotherapy and for timely implementation of new therapeutic strategies.

Response Evaluation Criteria in Solid Tumors (RECIST) are the standard most often utilized to evaluate tumor response to chemotherapy, which relies on morphological measurements of tumor size based on CT or MRI examination [2]. However, reduction in tumor size often takes several weeks or months to be measurable after initiation of treatment. Functional imaging modalities have been used to detect alterations in the perfusion or metabolism of the tumors and often provide valuable information earlier during therapy [3–5]. However, these imaging techniques are variously limited by their cost, the patient's risk of radiation exposure, and the need for contrast agents.

Changes in tumor metabolism, perfusion, receptor expression, and survival (apoptosis and/or necrosis) are common responses to chemotherapy that occur far earlier than changes in tumor volume [6]. Cancer cell death in the early stage of therapy has been demonstrated to be an effective indicator to predict treatment outcome in both preclinical and clinical studies [7, 8]. Currently, histological analysis is the standard methods to detect cancer cell death, however, this method needs to acquire tissue samples through biopsy which is invasive. Since effective treatment would lead to the reduction in cancer cell density, water apparent diffusion coefficient measured by diffusion-weighted magnetic resonance imaging (DW-MRI) has been showed to be increased in the treatment responder in clinical studies [9, 10]. However, in order to evaluate tumor response to treatments, repeated and dynamic examinations are required which limits the clinical utilization of DW-MRI since the cost of MRI is relative high. Comparing with other imaging technique using in assessing tumor response to chemotherapy, ultrasonography has the advantages as no ionizing radiation, easy availability, portability and relative low cost of ultrasound equipment. Based on the analysis of the ultrasonic radiofrequency (RF) data, quantitative analysis of the ultrasonic spectrum has been employed to collect information of tissue microstructures. In previous studies, this noninvasive technique has been used to diagnose some disease such as the anisotropy of myocardial structures, ocular tumors, and prostate cancer and to evaluate cancer cell apoptosis caused by anticancer therapies in mice tumor models [11–16]. Our previous study showed that quantitative analysis of the ultrasonic spectrum could be used to evaluate tumor response to cytotoxic therapy by characterizing tumor microstructure changes [17]. Unlike ultrasonic spectral analysis of a single frame, ultrasound RF time series is calculated through analyzing sequence of RF echoes continuously acquired from one stationary tissue location within a few seconds. Previous studies showed that RF time series had higher accuracy, sensitivity, and specificity in tissue typing in comparison with spectrum analysis of only one frame of RF data [18]. Past research also showed that when a location in the tissue receives repetitive irradiation of sequential ultrasound, the signal of RF time series backscattered from the location would carry information regarding “tissue characteristic” [19], and that changes in the magnitude of RF signal over time in the sample location depend on tissue microstructure [20]. Although the mechanisms responsible for these effects are not fully understood, it has been shown that variations in ultrasound backscattering are related to changes in both ultrasound speed and tissue temperature which occurs in the course of the ultrasonic RF time series scanning procedure, and depend in turn on tissue properties [21]. Ultrasonic RF time series has been utilized for the diagnosis of breast and prostate tumors, and to monitor changes after tissue ablation, with promising preliminary results [22–24]. However, as far as we know, no study has so far been published addressing the application of ultrasonic RF time series in the evaluation of early tumor response to the treatment of cytotoxic agents. Therefore, this study aimed to assess whether ultrasonic RF time series could be used to assess early tumor response to conventional chemotherapy in a mice breast cancer models.

## RESULTS

### Assessment of tumor growth

**Figure 1:**
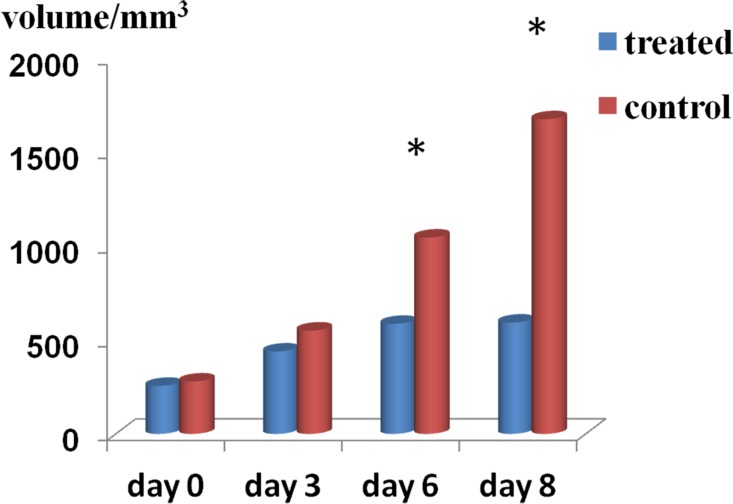
Tumor volume changes Summary bar graphs show that treatment with cisplatin and paclitaxel significantly reduced tumor growth on days 6 and 8 as compared with control tumors (^*^P < 0.01).

Tumor volume didn't significantly differ between control and chemotherapy-treated mice on days 0 and 3. On days 6 and 8, tumor volumes from treated mice were significantly smaller than control tumors (day 6: control group = 1.05 ± 0.38 cm^3^, treatment group = 0.59 ± 0.24 cm^3^, *P* < 0.01; day 8, control group = 1.68 ± 0.78 cm^3^, treatment group = 0.59 ± 0.23 cm^3^, *P* < 0.01) (Figure 1).

### Changes in ultrasonic RF time series features

**Figure 2:**
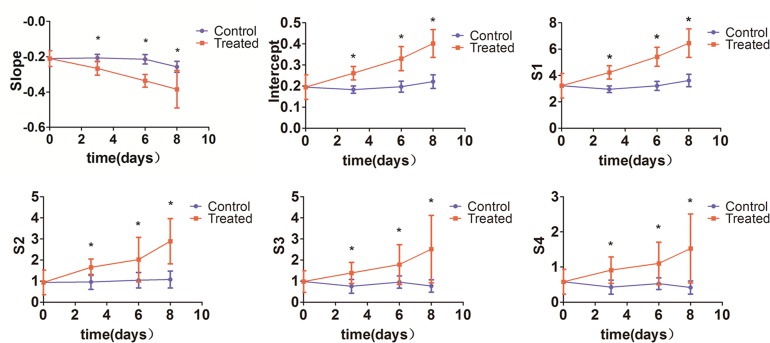
Changes in ultrasonic RF time series features *Intercept, S1, S2, S3 and S4* were significantly increased, and *slope* was significantly decreased in tumors from chemotherapy-treated mice compared with control tumors on days 3, 6, and 8 (^*^P < 0.01).

Significant differences were observed when comparing RF time series features of tumors from treated and control mice on days 3, 6, and 8 (*P* < 0.05). Significantly increased *intercept, S1, S2, S3*, and *S4* values, as well as decreased *slope*, were registered for treated tumors (*P* < 0.01) at those three time points (Figure 2). Compared to baseline (day 0), *intercept, S1*, and *S2* increased significantly, while the *slope* was significantly decreased (*P* < 0.01) on days 3, 6, and 8 in the treatment group. In contrast, no significant changes occurred in the 6 ultrasonic spectral features on days 3, 6, and 8 in the control group. We noticed that RF time series features demonstrated significant variations on day 3 after treatment, namely 3 days before differences in tumor size could be readily measured by conventional imaging.

### Histological changes

**Figure 3:**
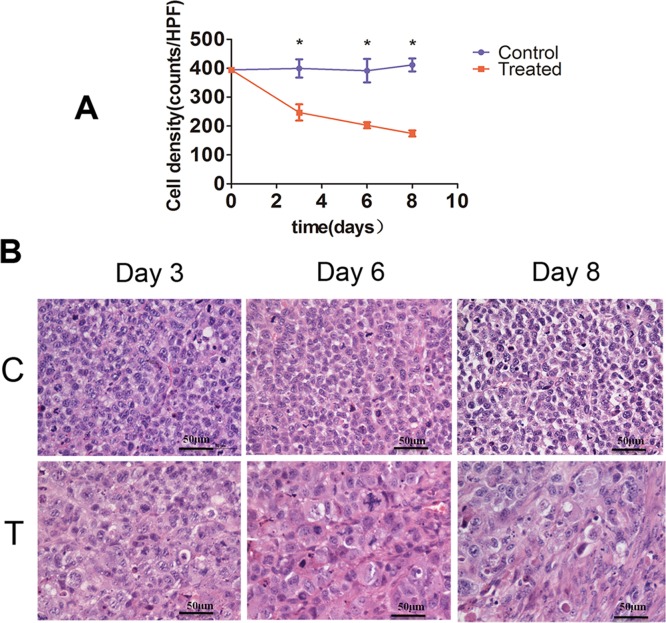
Histopathological analysis of tumor cell density **(A)** Bar graph summarizing changes in tumor cell density in control and treatment groups. (^*^P < 0.01). **(B)** Representative tissue micrographs showing substantially decreased tumor cell density in samples from chemotherapy-treated mice (T) as compared with control mice (C) receiving vehicle on days 3, 6, and 8. (Scale bars: 50 μm).

In the control tumors, tumor cell density did not significantly differ on days 3, 6, and 8 in comparison with day 0, while in samples from the treated tumors, treatment with cisplatin and paclitaxel significantly decreased tumor cell density on days 3, 6, and 8 relative to day 0 (*P* < 0.05). Compared with control, chemotherapy treatment significantly reduced tumor cell density on days 3, 6, and 8. Additional microstructural changes, involving nuclear condensation and fragmentation were also revealed by H&E staining in tumor specimens from chemotherapy-treated mice (Figure 3).

### Correlation between ultrasonic RF time series features and histological results

**Figure 4:**
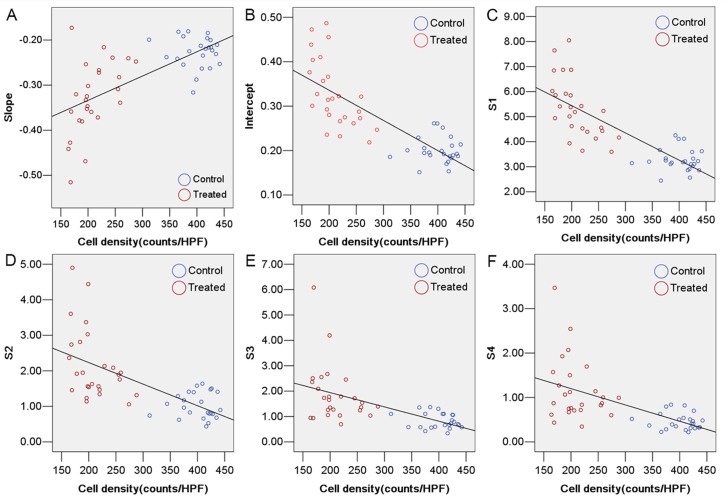
Correlation between tumor cell density and ultrasonic RF time series features **(A)** *slope* (r = 0.67, *P* < 0.01). **(B)** *intercept* (r = −0.84, *P* < 0.01). **(C)** *S1* (r = −0.84, *P* < 0.01). **(D)** *S2* (r = −0.70, *P* < 0.01). **(E)** *S3* (r = −0.66, *P* < 0.01). **(F)** *S4* (r = −0.67, *P* < 0.01).

A negative correlation was observed between tumor cell density and *intercept* (r = −0.84, *P* < 0.01)*, S1* (r = −0.84, *P* < 0.01)*, S2* (r = −0.70, *P* < 0.01), *S3* (r = −0.66, *P* < 0.01), and *S4* (r = −0.67, *P* < 0.01). In contrast, there was positive correlation between tumor cell density and *slope* (r = 0.67, *P* < 0.01) (Figure 4).

## DISCUSSION

This study was designed to evaluate the feasibility of ultrasonic RF time series in the assessment of early tumor response to combination chemotherapy with cisplatin and paclitaxel in a subcutaneous breast cancer model in mice. With the use of tumor size and tumor cell nuclei density as end-point measurements, our results revealed that ultrasonic RF time series data showed good correlation with longitudinal treatment response following chemotherapy. Whereas conventional imaging could detect the differences in tumor size by day 6, changes in ultrasound RF time series parameters were evident just 3 days after treatment. Paralleling these changes, histopathology revealed significant chemotherapy-induced alterations in tumor microstructure.

Early monitoring of tumor response to anticancer therapy is crucial to prevent further unnecessary therapy and to help determining new treatment choices. For a long time, the Response Evaluation Criteria in Solid Tumors (RECIST), which relies on CT- or MRI-based morphological measurements, has been widely applicated for evaluation of clinical responses [2, 25]. However, since the tumor shrinkage occurs late in the course of treatment, it is hard to be determined early tumor response to chemotherapy based on conventional radiographic modalities [26]. Therefore, there is a strong need to develop and validate reliable imaging techniques for this purpose.

Cancer cell death in the early stage of therapy has been demonstrated to be an effective indicator to predict treatment outcome in both preclinical and clinical studies [7, 8]. Owing to tumor cell death, tumor cell density would significantly decrease after treatment [27] and this reduction has become a hallmark of early tumor response to chemotherapy [6]. In this study, the decrease in tumor cell density was the most significant changes in tumor microstructure after treatment, which could be detected just 3 days following treatment initiation. Previous clinical studies showed that these changes were also found in breast cancer which was response to neoadjuvant chemotherapy [27, 28]. Due to the decrease in tumor cell density, apparent diffusion coefficient of water measured by diffusion-weighted MRI has been shown to be increased in patients with breast [29, 30] and ovarian cancers which was response to chemotherapy [10], soon after treatment (within 2–4 days) and before changes in tumor volume could be measureable. However, the application MRI in assessing tumor response to chemotherapy is limited due to its relative high cost.

Ultrasound imaging offers some major advantages, i.e. relatively low cost, wide availability and portability, and no risk of exposing to radiation in comparison to other imaging modalities utilized to assess tumor response to treatment. Ultrasound have been used for tissue characterization since the early 70's by quantitatively analyzing the RF signal backscattered from the tissue [31]. The usefulness of ultrasonic spectrum analysis of single RF frame data in assessing tumor response to radiotherapy [15] and chemotherapy [32] has been investigated by parallel analyses of tissue microstructural changes. RF time series analysis, which is calculated through analyzing sequence of RF echoes continuously acquired from one stationary tissue location within a few seconds, has been proposed as a new tissue characterizing technique for tissue typing to enable cancer detection (18, 21, 23). Although both features extracted from RF time series and features of spectrum analysis are calculated from the ultrasound RF signals, the principles underlined above two means are different, as features of spectrum analysis are computed from only one single frame of RF data, while the features of RF time series are calculated from sequence of ultrasound RF echoes that originate from a constant tissue location and depth [18]. Hence, an advantage of RF time series analysis over single-frame spectrum analysis is that in the former there is no need for signal compensation to account for depth-dependent effects [33].

Ultrasound beams undergo scattering and will be absorbed by tissue when propagating through tissue. Moradi and his colleagues proposed that when a location in the tissue receives repetitive irradiation of sequential ultrasound, the signal of RF time series backscattered from the location would carry information regarding “tissue characteristic”. Their study showed that compared to traditional ultrasonic spectral features and texture analysis of B-scan, RF time series was superior in detecting prostate cancer. The area under the receiver operating characteristic curve of RF time series was 0.87, which was significantly higher than traditional ultrasonic spectral analysis (0.78) and the texture analysis (0.72) [18]. The potential reasons underlining the improved performance might be due to the changes in temperature and sound speed caused by continuous sonification of the frame sequences [22]. The absorption of the mechanical energy of the ultrasound beams in the tissue will induce thermal effects and potentially cause a small increase in the temperature, which will change the ultrasound speed and induce a time shift in receiving the ultrasound signal backscattering from the tissue. The increase in the temperature due to ultrasound propagation depends on the thermal properties of tissue and varies from one to another [22].

Our study has several limitations. For instance, it was hard to exactly match the imaging plane on ultrasound system with the pathological section since the ultrasound transducer is much thicker than the histopathological section. However, as tumor microstructures seen on HE staining were mostly homogenous, this should have not led to significantly bias. Quantification of the ultrasonic features with three dimensional transducers which covered the whole tumor area could improve the definition of the features calculated from the sequential RF data to better correlate them with concomitant pathological changes. Second, ultrasound attenuation during propagating in the tissue was not made up for in the current study since tumors were located subcutaneously and we placed the ROI center at about 0.2 cm under the skin. It was reasonable to think the results would not be significantly changed even if the ultrasound attenuation of a 6 MHz transducer was compensated. Third, no comparison was made between ultrasonic RF time series and single frame spectrum analysis, although previous studies had showed that ultrasonic RF time series had higher accuracy, sensitivity, and specificity in tissue characterization compared with ultrasonic spectrum analysis of a single RF frame (18, 23, 33). Nevertheless, further studies are needed to fully characterize and compare the performance of these two imaging modalities during early assessment of chemotherapy response.

In conclusion, our preclinical study suggested that ultrasonic RF time series offered a simple way to noninvasively detect early tumor microstructure changes post chemotherapy with the use of a clinical ultrasound system. In line with previous reports, we propose that ultrasonic RF time series could be utilized for early evaluation of tumor response to conventional chemotherapy before changes in tumor volume become detectable without contrast agent injection.

## MATERIALS AND METHODS

### Cell line and tumor implantation

This study was approved by the Committee on the Ethics of Animal Experiments of the Sun Yat-Sen University and followed the Guide for the Care and Use of Laboratory Animals of the National Institutes of Health. The human breast cancer cell line MCF-7 was obtained from the State Key Laboratory of Oncology in Southern China. Cells were grown in DMEM (HyClone Co., UT, USA) supplemented with 10% fetal bovine serum (Gibco, Grand Island, NY, USA), penicillin (50 U/ml), and streptomycin (50 μg/ml) at 37°C in a humidified 5% CO_2_ atmosphere. For inoculation, approximately 5 × 10^7^ MCF-7 cells suspended in phosphate-buffered saline were injected subcutaneously into the right chest wall of 5- to 6-week-old BALB/c female nude mice.

### Experimental design

**Figure 5:**
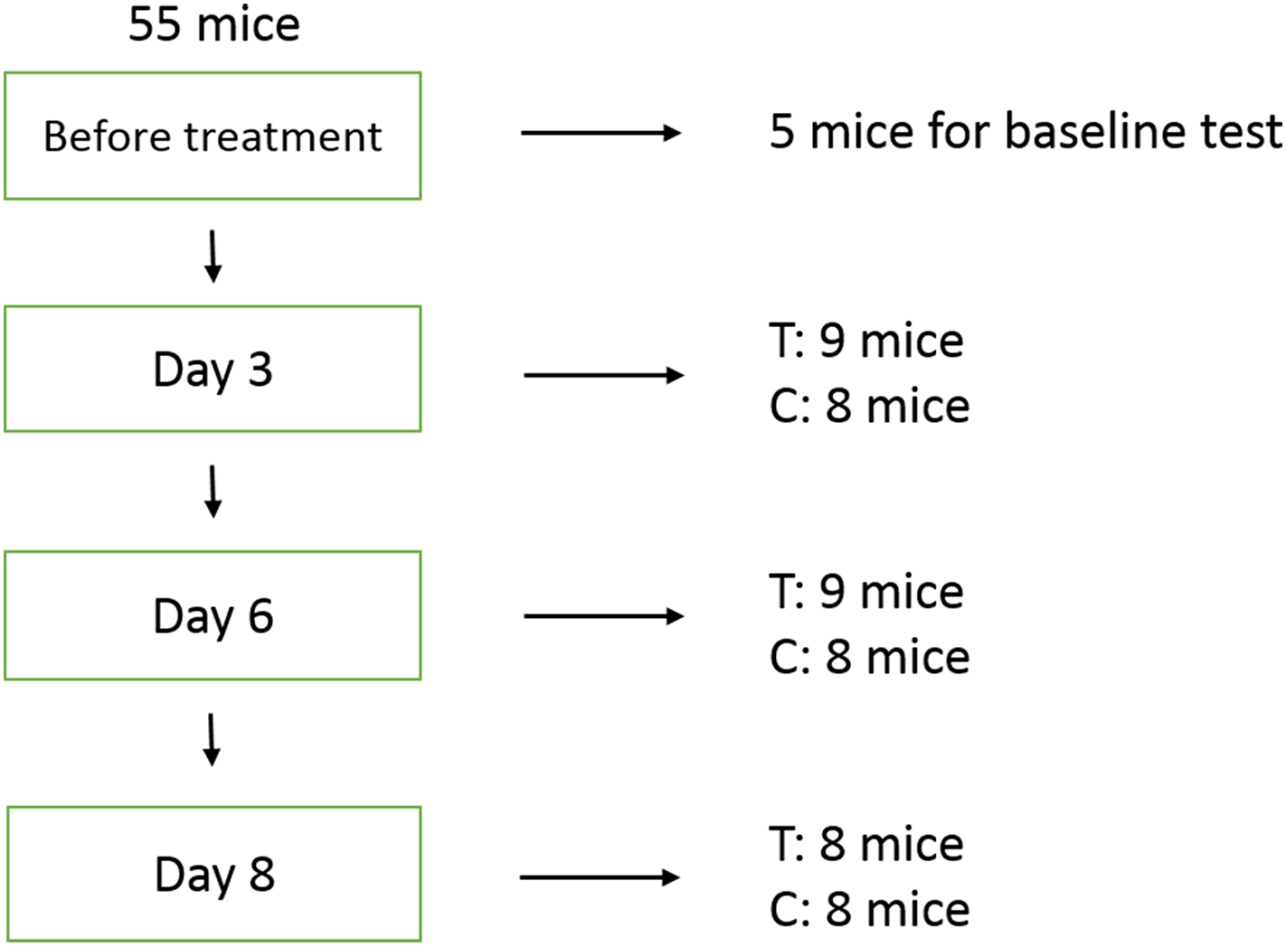
Experimental design Five mice were randomly chosen for baseline evaluation prior to treatment. On days 3 and 6, 8 mice from the control group and 9 mice from the treatment (chemotherapy) group were sacrificed after ultrasound imaging and tumors were excised for histopathologic analysis. The same procedures were performed on day 8 on the remaining 16 mice (8 mice per group). T: treatment; C: control.

A total of 55 mice were used for the experiments. After implantation MCF-7 tumors were allowed to grow for 14 days, until their longest diameter reached ~8-10 mm. The first-dose chemotherapy time point was referred to as day 0. At this point, 5 untreated mice were randomly chosen for ultrasonic examination, and their tumors then excised for histopathological analysis. The remaining mice were randomized into a treatment group (n = 26) and a control group (n = 24). The mice in the treatment group received combination chemotherapy with cisplatin (2 mg/kg.d^-1^, Mayne Pharma Pty Ltd, Salisbury, Australia) and paclitaxel (10 mg/kg.d^-1^, Bristol-Myers Squibb, Italy) by intraperitoneal injection once daily for 3 days. The mice in the control group received vehicle (sterile saline) with the same timing and dosing schedule. After the last dose, ultrasound imaging was performed on days 3, 6, and 8. On days 3 and 6, 8 mice from the control group and 9 mice from the treatment group were sacrificed after ultrasound imaging and tumors were excised for histopathological analysis. On day 8, the same procedures were carried out in the remaining 16 mice (8 mice per group) (Figure 5).

### Ultrasound data acquisition

Ultrasound imaging was performed on days 0, 3, 6, and 8. Each mouse was anesthetized by intraperitoneal injection of pentobarbital sodium at a dose of 75 mg/kg (Sigma, St. Louis, MO, USA) before imaging. Centrifuged gel was used to minimize bubble formation in the gel and a stand-off gel pad was placed on the skin for scanning. A commercially available clinical ultrasound scanner, Sonix TOUCH (Ultrasonix Medical Corporation, Richmond, Canada) with an L14–5/38 ultrasound transducer at a center frequency of 6 MHz was used to simultaneously collect B-mode tumor images and RF data. For data acquisition, the ultrasound transducer was positioned such that the focal zone was at the same depth in each imaged specimen to control for any potential attenuation. All RF data were sampled with mechanical index of 0.25, frame rate of 33 Hz, dynamic range of 76 dB, and imaging depth of 2.5 cm. Imaging settings, including TGC, gain, power, and sampling rate were identical in all imaging sessions. The ultrasound probe and the tumors remained stationary in position for 10 seconds and a total of 330 RF data frames were digitally recorded at three different image planes for each tumor. All ultrasound examinations were performed by one radiologist (J.W.W), who was blinded to treatment status. The greatest longitudinal, transverse, and anteroposterior dimensions of tumors were registered each time by electronic caliper measurements available on the ultrasound system. Tumor volume was determined using the formula for a prolate ellipsoid: volume = π/6 × length × width × depth. The largest cross-section plane of the tumor was imaged with the transducer held manually in this position throughout the examination.

### Features extraction from RF time series

Ultrasound RF time series analysis was performed by a radiologist (L.Q.G) who was blinded to treatment information. All the ultrasound RF data was analyzed with MATLAB-based (v. 2009a: MathWorks, Natick, MA, USA) software developed in our lab for ultrasound RF time series analysis. Rectangular regions of interest (ROI) were centered approximately at the focal depth of the transducer onto tumor images. Three representative ROIs were selected for each tumor sample and averaged for the final analysis.

**Figure 6:**
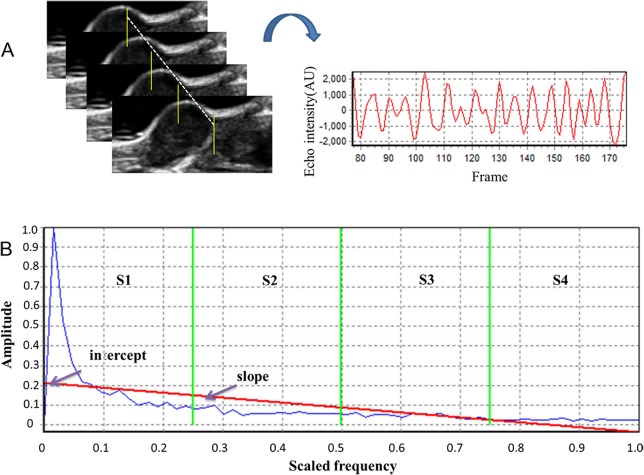
Analysis of ultrasonic RF time series features **(A)** Samples of RF echo signals collected over time from a fixed tumor location under continuous emission of ultrasound. **(B)** Quantitative features included the *slope* and *intercept* of the linear regression of the frequency spectrum (red line), and the sum of the amplitude values in four different frequency bands (separated by green vertical lines on the graphs), namely *S1-S4*.

Temporal ultrasound RF echo signals collected from a fixed spot of tumor tissue formed one RF time series (Figure 6A). We use the method originally described by Moradi et al. which proposed summarizing the power spectrum of the RF time series in six features, as described below [21]. These six features were extracted from the amplitude of the Discrete Fourier Transformation (DFT) of RF time series averaged over an ROI. For analysis, a window of size M×N was selected to form RF time series. The power spectrum was averaged over the ROI and then normalized by dividing it by its maximum to obtain values in the range [0, 1]. This normalization process sets the maximum of the averaged spectrum to 1 and enables us to compare data from different ROIs. As all samples of one RF time series originated from the same depth of the tissue, there was no need for compensation of signals for depth-dependent effects. The normalized amplitude of the DFT can be described as:

$$f_{i}^{\text{nor}}=\frac{{f^{\prime }}_{i}}{\frac{1}{N_{\text{t}}}\sum_{j=1}^{N_{t}}\left|{f^{\prime }}_{j}\right|},{f^{\prime }}_{i}=f_{i}-\frac{1}{N_{t}}\sum_{j=1}^{N_{t}}f_{j},i=1,2,\cdots ,N_{t}.$$ (1)

where *f_i_* is the amplitude of RF time series in point *i*, and *N_i_* is the amount of RF time series. We transform the RF time series *f_i_* into *F(k)* using the Fast Fourier Transform (FFT) algorithm as implemented in MATLAB. Then we average the spectrum over all RF time series corresponding to RF samples in one ROI. The averaged spectrum of the ROI (*F_ave_*(*k*)) is then normalized as follows:

$$|F_{\text{ave}}(k)|=|F_{\text{ave}}(k)|/\max\left(\left|F_{\text{ave}}(k)\right|\right)$$ (2)

This normalization process sets the maximum of the averaged spectrum to 1 and enables us to compare data from different ROIs. The six proposed RF time series features, listed below, are extracted from ${\hat{F}}_{\text{ave}}(k)$ and are designed to represent the frequency spectrum through only a few parameters. The first four features (*S1, S2, S3*, and *S4*) are the integral of ${\hat{F}}_{\text{ave}}(k)$ in four quarters of the frequency range:

S1=∑k=1L8|Fave(k)|,S2=∑k=L8+1L4|Fave(k)|S3=∑k=L4+134L|Fave(k)|,S4=∑k=3L4+1L2|Fave(k)| (3)

where *L* is the length of the RF time series. We also fit a regression line to values of the spectrum versus normalized frequency. The *slope* and *intercept* of the regression line are the remaining two features (Figure 6B).

### Histopathological examination

At each time point after ultrasound imaging, mice were sacrificed and tumors were removed and fixed in 10% buffered formalin before paraffin processing. Tumor specimens were sectioned (5 μm) at the largest cross sections corresponding to the ultrasound imaging planes. Sections were stained with hematoxylin and eosin (H&E) and assessed microscopically for changes in cell morphology. Tumor cell density was measured by a team member (L.Q) who was blinded to treatment status. Regions with the highest tumor cell density in H&E stained sections were located by scanning the sections under 40 × magnification optics, and ten different fields within such regions were randomly captured at 400 × magnification. Image-Pro Plus 6.0 software (Media Cybernetics, Silver Spring, MD, USA) was used to calculate the number of nuclei in each histology image. Data were averaged over ten fields for statistical analysis.

### Statistical analysis

All analyses were performed using SPSS version 16.0 (SPSS, Inc, Chicago, IL). The Kolmogorov-Smirnov test was applied to evaluate normal distribution. The Levene test was applied to evaluate the homogeneity of variance. Independent student's t-test was used to assess significance when comparing tumor sizes between treated and control mice. One-way analysis of variance (ANOVA) was used to determine whether significant differences in RF time series features and tumor nuclei density existed at the three imaging points between the treatment and control groups. The post-hoc Bonferroni corrected t test was performed for multiple comparisons to confirm differences between individual time points. The Pearson correlation test was used to determine the relationship between RF time series features and histopathological changes. *P* ≤ 0.05 was considered as being statistically significant.
